# Hepatitis C virus treatment for prevention among people who inject drugs: Modeling treatment scale-up in the age of direct-acting antivirals

**DOI:** 10.1002/hep.26431

**Published:** 2013-08-26

**Authors:** Natasha K Martin, Peter Vickerman, Jason Grebely, Margaret Hellard, Sharon J Hutchinson, Viviane D Lima, Graham R Foster, John F Dillon, David J Goldberg, Gregory J Dore, Matthew Hickman

**Affiliations:** 1 School of Social and Community Medicine, University of BristolBristol, UK; 2 Social and Mathematical Epidemiology Group, Department of Global Health and Development, London School of Hygiene and Tropical MedicineLondon, UK; 3 Kirby Institute, University of New South WalesSydney, Australia; 4 Burnet InstituteMelbourne, Australia; 5 Health Protection ScotlandGlasgow, UK; 6 School of Health and Life Sciences, Glasgow Caledonian UniversityGlasgow, UK; 7 British Columbia Centre for Excellence in HIV/AIDS, University of British ColumbiaVancouver, Canada; 8 Blizard Institute, Queen Mary's University of LondonLondon, UK; 9 Medical Research Institute, Ninewells Hospital, University of DundeeDundee, UK

## Abstract

Substantial reductions in hepatitis C virus (HCV) prevalence among people who inject drugs (PWID) cannot be achieved by harm reduction interventions such as needle exchange and opiate substitution therapy (OST) alone. Current HCV treatment is arduous and uptake is low, but new highly effective and tolerable interferon-free direct-acting antiviral (DAA) treatments could facilitate increased uptake. We projected the potential impact of DAA treatments on PWID HCV prevalence in three settings. A dynamic HCV transmission model was parameterized to three chronic HCV prevalence settings: Edinburgh, UK (25%); Melbourne, Australia (50%); and Vancouver, Canada (65%). Using realistic scenarios of future DAAs (90% sustained viral response, 12 weeks duration, available 2015), we projected the treatment rates required to reduce chronic HCV prevalence by half or three-quarters within 15 years. Current HCV treatment rates may have a minimal impact on prevalence in Melbourne and Vancouver (<2% relative reductions) but could reduce prevalence by 26% in 15 years in Edinburgh. Prevalence could halve within 15 years with treatment scale-up to 15, 40, or 76 per 1,000 PWID annually in Edinburgh, Melbourne, or Vancouver, respectively (2-, 13-, and 15-fold increases, respectively). Scale-up to 22, 54, or 98 per 1,000 PWID annually could reduce prevalence by three-quarters within 15 years. Less impact occurs with delayed scale-up, higher baseline prevalence, or shorter average injecting duration. Results are insensitive to risk heterogeneity or restricting treatment to PWID on OST. At existing HCV drug costs, halving chronic prevalence would require annual treatment budgets of US $3.2 million in Edinburgh and approximately $50 million in Melbourne and Vancouver. *Conclusion*: Interferon-free DAAs could enable increased HCV treatment uptake among PWID, which could have a major preventative impact. However, treatment costs may limit scale-up, and should be addressed. (Hepatology 2013;58:1598–1609)

The global burden of hepatitis C virus (HCV) infection continues to rise.^1^,^2^ The core of the HCV epidemic in the developed world occurs among people who inject drugs (PWID), who comprise the majority of new (80%) and existing (60%) cases.^1^ Globally, HCV seroprevalence (>60% in most countries)^3^ and incidence (5%-40% annually)^4^,^5^ remains high among PWID. Prevention strategies, such as needle and syringe programs (NSP) and opiate substitution therapy (OST), can reduce HCV transmission and have maintained low levels of human immunodeficiency virus (HIV) infection in many settings, but they are insufficient to achieve substantial reductions in HCV prevalence.^6^–^9^ This is partly because high HCV prevalence and long injecting duration among PWID in many settings combine such that the intervention coverage required for major prevalence reductions is unobtainable and unsustainable.^9^ Given that there is no HCV vaccine, alternative strategies for HCV prevention are urgently needed.

In HIV, the demonstration that antiretroviral therapy given to HIV-infected individuals can prevent secondary transmission has generated considerable excitement^10^ and suggests that we may have reached a tipping point for preventing HIV transmission.^11^ In contrast to HIV, HCV is curable and therapy is finite. Therefore, HCV treatment as prevention may provide even greater opportunity for preventing onward HCV transmission and directly reducing HCV chronic prevalence.

Mathematical modeling studies have suggested HCV treatment for PWID could be an effective^12^–^16^ and cost-effective^17^ intervention to prevent HCV transmission. However, these studies only considered treatment with pegylated interferon (PEG-IFN) and ribavirin (RBV). The feasibility of expanding this treatment regimen as a strategy for treatment as prevention is limited, given the poor tolerability and limited uptake of PEG-IFN+RBV therapy, particularly among PWID.^18^,^19^ However, therapeutic options for HCV are evolving rapidly. Preliminary data from IFN-free direct-acting antiviral (DAA) therapy phase 2 trials indicates that in the near future, regimens will be available with markedly reduced toxicity, high efficacy (>90% cure), improved dosing schedules (once or twice-daily), and shortened treatment duration (6-24 weeks).^20^–^22^ Such advances indicate that a HCV treatment as prevention strategy among PWID may be feasible in the very near future.

We project the potential impact of DAA therapy on HCV prevalence in three international settings with varied prevalence.

## Subjects and Methods

### Mathematical Model

**Figure 1:**
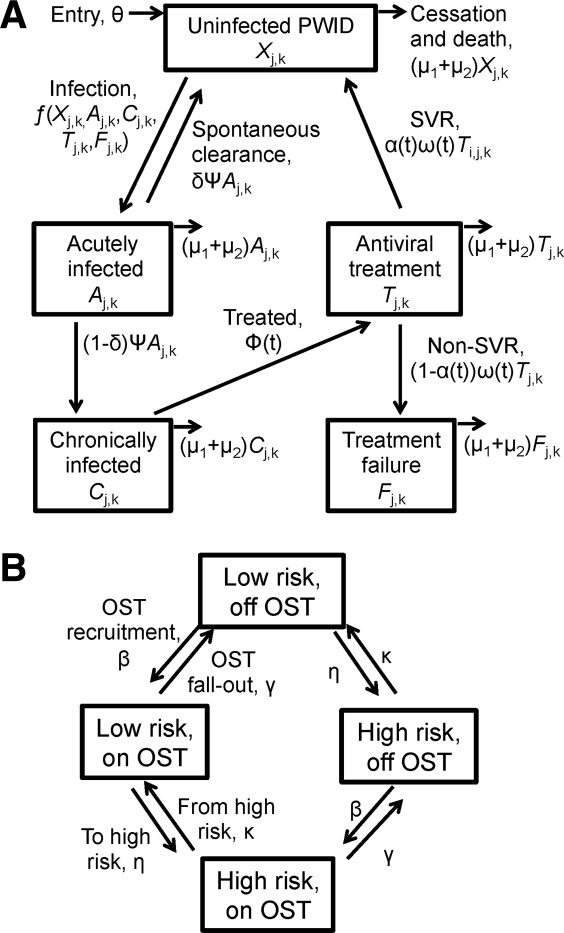
Model schematic showing HCV disease transmission and treatment states (A) and behavioral states (B). (A) Compartments for uninfected PWID (*X_j__,__k_*), acutely infected PWID (*A_j__,__k_*), chronically infected PWID (*C_j__,__k_*), PWID on antiviral treatment (*T_j__,__k_*), and PWID treatment failures (F*_j__,__k_*). (B) The population was stratified by risk (low/high, *j* = 0 or 1, respectively), and OST (off/on, *k* = 0 or 1, respectively). New PWID enter the model at a constant rate (θ) as uninfected, off OST, and either low or high risk. Uninfected PWID can become acutely infected with HCV, where a proportion (δ) of individuals spontaneously clear their acute infection after a duration of time (1/ψ), and return to the uninfected compartment. Those who do not spontaneously clear the acute infection (1-δ) progress to chronic infection, where they are eligible for antiviral treatment. Because PWID are unlikely to be diagnosed during acute infection, it was assumed that they are not treated during the acute stage. If treated, a proportion [α(t)] achieve SVR and return to the uninfected compartment. Those who do not attain SVR [1-α(t)] move to the treatment failure compartment, where they cannot be retreated. PWID exit all compartments due to permanent cessation of drug use (μ_1_) or death due to drug or non–drug-related causes (μ_2_). The base-case analysis assumed PWID transition between high/low risk stages, as well as on/off OST. Additional details are provided in the Supporting Information.

A deterministic HCV transmission and treatment model among PWID^12^ was extended to incorporate additional biological and behavioral complexity (details in Fig. 1 and Supporting Information). The modeled population was stratified by infection state (uninfected, acute HCV, chronic HCV, on antiviral treatment, treatment failure), transmission risk (low/high), and current OST status (on/off). A fixed number (Φ(t)) of chronically infected PWID initiate treatment annually (or all chronic infections if fewer than Φ(t) are chronically infected), for a treatment duration of 1/ω(t). It was conservatively assumed treatment failures (those who do not attain sustained viral response [SVR]) could not be retreated due to potential resistance and reluctance to undergo further therapy. Furthermore, at baseline, few IFN+RBV treatment failures exist due to historically low treatment rates for PWID. In the base-case, PWID who are low-/high-risk and on/off OST are eligible for treatment; restricted treatment for only low-risk or those on OST was explored in the sensitivity analysis.

Because the model is dynamic, the risk of infection or reinfection for a PWID is proportional to HCV prevalence, which changes over time. We do not assume any risk difference after treatment; reinfection risk is equal to primary infection risk. The forces of infection for each susceptible state were defined by the relative risk in that state, such that infectivity and susceptibility were altered by a factor Γ, Π, or Γ × Π if the PWID was on OST, high risk, or both, respectively. This was assumed to occur through a corresponding change in the relative frequency of transmission events with other PWID. The chance of a PWID having a transmission event with any PWID from another risk state and infectious status was proportional to the relative frequency of transmission events for PWID in that state. Due to rapid reductions of HCV RNA levels during treatment,^23^ we assumed the proportion on treatment who eventually achieve SVR (α(t)) are not infectious, whereas the remainder (1-α(t)) remain infectious. Some evidence indicates that acute infection may be associated with 2-log higher viral loads than during chronic infection^24^; however no studies have shown increased transmissibility during this stage. Therefore, we assumed equal infectivity for the base-case, but considered a five-fold higher transmissibility during acute HCV in the sensitivity analysis (assuming a similar relationship between viral load and transmissibility as for HIV^25^).

### Modeling Treatment Scale-Up and Regimes

Treatment rates, durations, and SVR were varied over time to model scale-up and new treatments (see Supporting Information). No treatment prior to 2002 was modeled, because clinical guidance recommended against treatment of PWID. Due to the lack of reliable treatment data before 2007, a linear scale-up to current baseline treatment rates during 2002-2007 was modeled, with baseline rates constant during 2007-2012. Prior to 2012, we assumed all treatments used PEG-IFN+RBV. We assumed a continuation of baseline treatment rates from 2012-2015, during which time triple therapy with PEG-IFN+RBV and telaprevir/boceprevir will be available,^22^ although due to potential contraindications/drug interactions among PWID, we assumed only half of genotype 1 patients would be eligible for triple therapy. IFN-free DAAs were assumed to become available in 2015, followed by a 2-year linear scale-up in treatment (2015-2017) to scaled-up treatment rates (implemented from 2017-2027).

**Figure 2:**
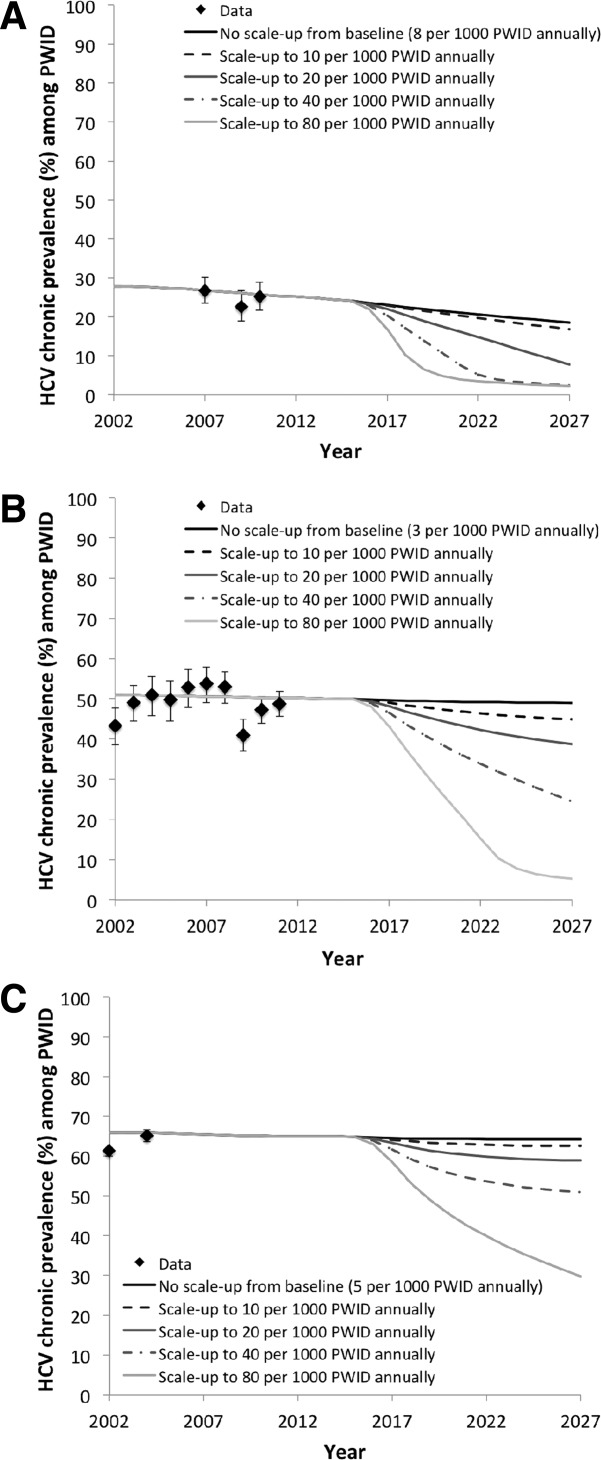
Chronic prevalence over time in (A) Edinburgh, (B) Melbourne, and (C) Vancouver. Simulations show no scale-up from baseline, or scale-up to 10, 20, 40, or 80 per 1,000 PWID treated annually. We assume no treatment prior to 2002, a linear scale-up to baseline treatment rates during 2002-2007, and baseline treatment rates during 2007-2012. A linear scale-up from baseline to scaled-up rate during 2015-2017 was modeled. HCV prevalence data points shown for comparison with 95% confidence intervals.

### Multivariate Uncertainty Analyses

To consider the effect of uncertainty in the underlying parameters, we performed a multivariate probabilistic uncertainty analysis where 1,000 parameter sets were randomly sampled from setting specific parameter distributions in Table1. For each of the 1,000 parameter sets, the model was calibrated to the sampled HCV chronic prevalence in 2012 and proportion on OST/high risk by varying π, β, and η. The model was then used to project the prevalence reductions in each setting over 15 years (2012-2027) with no treatment scale-up, or scale-up to rates of 10, 20, 40, or 80 per 1,000 PWID annually. Additionally, the required scaled-up treatment rates to achieve prevalence reductions of ¼, ½, or ¾ within 15 years were projected. For all projections, 95% credibility intervals (CrI) were generated from the multivariate uncertainty sampling. A linear regression analysis of covariance was performed on the 15-year impact with scale-up to 10 per 1,000 PWID annually, and the proportion of the sum-of-squares contributed by each parameter was calculated to estimate the importance of individual parameters to the overall uncertainty.

**Table 1:** Model Parameters and Sources

		Point Value (Sampling Bounds)				
Parameter	Symbol	Edinburgh, UK	Melbourne, Australia	Vancouver, Canada	Units	References
HCV chronic prevalence among PWID in 2012*	Vary π to fit	25% (22%-28%)	50% (47%-53%)	65% (63%-67%)	—	Edinburgh: Health Protection Scotland and the University of the West of Scotland,^26^ University of the West of Scotland, Health Protection Scotland, and West of Scotland Specialist Virology Centre^36^
						Melbourne: Iverson and Maher^19^
						Vancouver: Grebely et al.,^18^ Kim et al.^28^
						Sampled from a uniform distribution
PWID population size (used to calculate baseline treatment rate)†		4,240	25,000	13,500	—	Edinburgh: Hay et al.^51^ (∼0.9% of population)
						Melbourne: Iverson and Maher,^19^ Kirwan et al.^52^ (∼0.6% of the population)
						Vancouver: McInnes et al.^53^ (∼2.1% of the population)
Baseline treatment rate (from 2007 onward)	Φ(t) for t ≥2007	8 (4-12)	3 (1.5-4.5)	5 (2.5-7.5)	Per 1,000 PWID per year	Edinburgh: Innes et al.,^54^ unpublished data
						Melbourne: Iverson et al.,^27^ unpublished data
						Vancouver: Grebely et al.,^18^ unpublished data
						Sampled from a uniform distribution
Proportion G1		53% (49%-57%)	56% (49%-63%)	60% (56%-64%)	—	Edinburgh: Innes et al.,^54^ unpublished data
						Melbourne: Aitken et al.,^55^ McCaw et al.^56^
						Vancouver: Grebely et al.,^18^ unpublished data
						Sampled from a uniform distribution
Death rate	μ_2_	1%	0.83%	3%	Per year	Edinburgh: Hickman et al.,^30^ Cornish et al.^31^
						Melbourne: Stoove et al.^32^
						Vancouver: Urban Health Research Initiative of the British Columbia Centre for Excellence in HIV/AIDS^33^
						Sampled from a Poisson distribution
Proportion PWID on OST	Vary β to fit	57% (50%-64%)	48% (44%-52%)	45% (43%-47%)		Edinburgh: University of the West of Scotland, Health Protection Scotland, and West of Scotland Specialist Virology Centre,^36^ unpublished data‡
						Melbourne: Iverson and Maher,^19^ Kirwan et al.^52^
						Vancouver: Urban Health Research Initiative of the British Columbia Centre for Excellence in HIV/AIDS^33^
						Sampled from a uniform distribution	
Duration on OST	12/γ	8 (4-12)	6.5 (3.25-8.75)	7 (3.5-10.5)	Months	Edinburgh: Cornish et al.^31^
						Melbourne: Burns et al.^57^
						Vancouver: Nosyk et al.^58^
						Sampled from a uniform distribution
Proportion PWID high risk§	φ, and vary η to fit	33% (27%-39%)	17% (14%-20%)	64% (62%-66%)	—	Edinburgh: University of the West of Scotland, Health Protection Scotland, and West of Scotland Specialist Virology Centre,^36^ Allen et al.^59^
						Melbourne: O'Keefe et al.,^60^ unpublished data
						Vancouver: Urban Health Research Initiative of the British Columbia Centre for Excellence in HIV/AIDS^33^
						Sampled from a uniform distribution
Duration high risk	12/κ	14 (7-21)	13 (6.5-19.5)	38 (19-57)	Months	Edinburgh: Vickerman et al.,^9^ Kemp et al.^61^
						Melbourne: O'Keefe et al.,^60^ unpublished data
						Vancouver: Urban Health Research Initiative of the British Columbia Centre for Excellence in HIV/AIDS,^33^ unpublished data
Proportion spontaneously clear	δ	26%	26%	26%	—	Micallef et al.^29^
Duration of acute stage	12/ψ	6 (3-9)	6 (3-9)	6 (3-9)	Months	Mondelli et al.^62^
Sampled from a uniform distribution						
Duration of injecting until final cessation	1/μ_1_	11 (6-20)	11 (6-27)	11 (6-23)	Years	Sweeting et al.,^34^ Kimber et al.^35^ (see Supporting Information for details)
						Sampled from a uniform distribution
SVR	α(t)∥					
PEG-IFN+RBV (G1)		37% (26%-48%)	37% (26%-48%)	37% (26%-48%)	—	Aspinall et al.^37^
						Sampled from a uniform distribution
PEG-IFN+RBV (G2/3)		67% (56%-78%)	67% (56%-78%)	67% (56%-78%)	—	Aspinall et al.^37^
	Sampled from a uniform distribution				
Telaprevir/Boceprevir (G1)		63% (44%-82%)	63% (44%-82%)	63% (44%-82%)	—	Jacobson et al.,^38^ Poordad et al.^39^
	Sampled from a uniform distribution				
IFN-free DAAs (all genotypes)		90%	90%	90%	—	Gane et al.,^20^ Poordad et al.,^21^ Dore^22^
	Estimated				
Treatment duration	52/ω(t)¶					
PEG-IFN+RBV (G1 SVR)		48	48	48	Weeks	National Institute for Health and Clinical Excellence^63^
PEG-IFN+RBV (G1)		12	12	12	Weeks	National Institute for Health and Clinical Excellence^63^
Non-SVR		24	24	24	Weeks	National Institute for Health and Clinical Excellence^63^
PEG-IFN+RBV (G2/3)		24#	24#	24#	Weeks	Jacobson et al.,^38^ Poordad et al.^39^
	Weighted estimate based on stopping rules				
Telaprevir/Boceprevir (G1)		12	12	12	Weeks	
IFN-free DAAs (all genotypes)						Dore^22^
	Estimated
Relative risk for acquiring HCV on OST	Γ	0.41 (0.21-0.82)	0.41 (0.21-0.82)	0.41 (0.21-0.82)	—	Turner et al.^8^
						Sampled from a lognormal distribution
Relative risk for high risk	Π	3.6 (1.5-8.7)	3.6 (1.5-8.7)	1.4 (1-2.1)	—	Edinburgh: Turner et al.,^8^ Allen et al.^59^
						Melbourne: Aitken et al.^55^ (assumed equal to Edinburgh)
						Vancouver: Kim et al.^28^
						Sampled from a lognormal distribution

* Used to estimate the infection rate, π (vary π and fit to the HCV chronic prevalence). Note that π is not the incidence rate.

† PWID population size was used to calculate baseline treatment rate per 1,000 PWID. Hence, for the model projections, the new injector entry rate (θ) was varied to fit to a total PWID population size of 1000.

‡ From 2008/2009 NESI survey excluding those who attended a survey recruitment site for methadone.

§ Defined as proportion PWID experiencing unstable housing.^8^,^28^,^55^,^59^

∥ When SVR rates vary by genotype, calculated using a weighted estimate based on population genotype distribution and SVR.

¶ When treatment durations vary by genotype, calculated using a weighted estimated based on genotype distribution, SVR, and treatment duration.

# Calculated based on early stopping rules and proportion achieving early viral response.

Abbreviations: G1, genotype 1; G2/3, genotype 2 or 3.

### Sensitivity Analysis

To evaluate the impact of individual model assumptions, univariate sensitivity analyses were performed on projected prevalence reductions at 15 years with a treatment rate of 10 per 1,000 PWID annually using the point parameter values in Table1. The analysis determined the impact of: delayed scale-up initiation (starting 2019 versus 2015); longer scale-up duration (6 versus 2 years); lower/higher DAA SVR (80%/100% versus 90%); increased infectivity during acute infection (five-fold infectiousness compared with chronic infection, equal in base-case); restricting treatment to only those on OST or low risk, shorter/longer average duration of injecting career (6/20 years versus 11 years); shorter/longer duration on OST; no turnover from high to low risk; greater differences between high/low risk (six times the relative risk between high/low risk versus two times); and changes in mixing behavior between high/low risk (fully assortative versus proportional).

### Model Parameterization

The model was parameterized to three international settings with a range of HCV chronic prevalence among PWID: Edinburgh, UK; Melbourne, Australia; and Vancouver, Canada. Model parameters and sources are given in Table1 and the Supporting Information.

HCV antibody prevalence estimates for Edinburgh, Melbourne, and Vancouver were 34%,^26^ 66%,^19^,^27^ and 88%,^18^,^28^ respectively. Because 26% of individuals spontaneously clear acute infection,^29^ it was assumed 74% of HCV antibody-positive individuals were chronically infected, resulting in HCV chronic prevalence estimates of 25% in Edinburgh, 50% in Melbourne, and 65% in Vancouver.

#### Death and Cessation Rates

PWID death rates were similar in Edinburgh (1% per year^30^,^31^) and Melbourne (0.83% per year^32^) but higher in Vancouver (3% per year^33^). Site-specific unbiased estimates of the average duration of injecting until cessation are unavailable and difficult to obtain. We assumed an average injecting duration of 11 years,^34^ but varied this from 6 years up to 20 or 27 years in the uncertainty/sensitivity analyses based on seroprevalence survey data.^19^,^28^,^35^,^36^

#### Baseline Treatment Rates

Current annual numbers treated and treatment rates were estimated as: 32 PWID annually (8 per 1,000 PWID) in Edinburgh, 75 PWID annually (3 per 1,000 PWID) in Melbourne, and 68 PWID annually (5 per 1,000 PWID) in Vancouver.

#### SVR Rates

SVR rates for PEG-IFN+RBV were obtained from a meta-analysis of treatment among PWID (37% [95% confidence interval, 26%-48%] for genotype 1; 67% [95% confidence interval, 56%-78%] for genotypes 2/3).^37^ Telaprevir/boceprevir with PEG-IFN+RBV increases genotype 1 SVR rates by 70% over PEG-IFN+RBV,^38^,^39^ so a 63% SVR rate was modeled. It was assumed IFN-free DAAs will achieve 90% SVR for all genotypes with a duration of 12 weeks.^20^–^22^

## Results

### Base-Case

**Figure 3:**
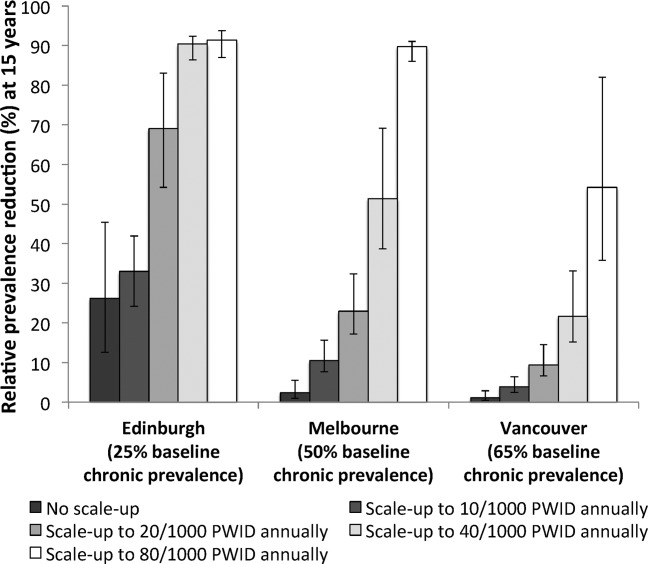
Relative prevalence reductions at 15 years (by 2027) with no treatment scale-up (8 per 1,000 PWID annually in Edinburgh, 3 per 1,000 PWID annually in Melbourne, and 5 per 1,000 PWID annually in Vancouver) and four treatment rate scenarios (10, 20, 40, and 80 per 1,000 PWID annually). Bars indicate the mean relative prevalence reductions, with whiskers representing the 95% CrI for the simulations.

Without any treatment scale-up, low chronic HCV prevalence in Edinburgh (25%) combined with switching to new DAAs and moderate baseline levels of treatment (8 per 1,000 PWID annually) could lead to a 26% (95% CrI, 13%-45%) relative reduction in prevalence within 15 years. However, in Melbourne and Vancouver, higher chronic HCV prevalence (50% and 65%, respectively) combined with low current levels of treatment (<5 per 1,000 PWID annually) produce little impact (<2%) on prevalence over 15 years. Figure 2 shows HCV chronic prevalence reductions over time, and Fig. 3 shows relative prevalence reductions at year 15 (10 years after full scale-up).

**Figure 4:**
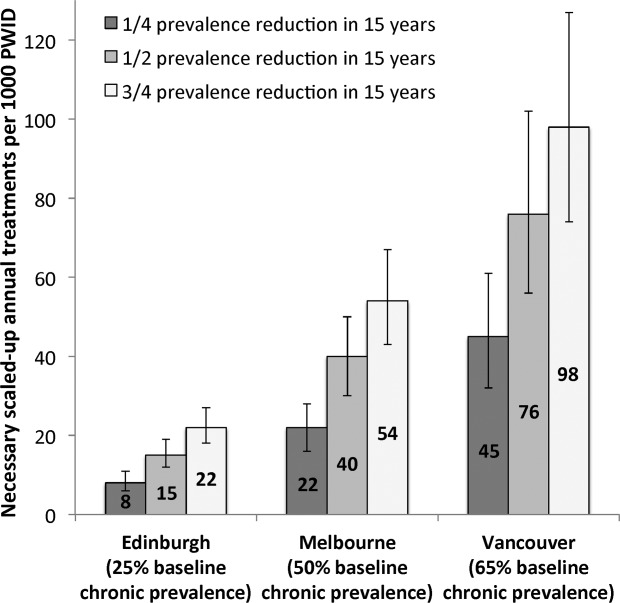
Annual scaled-up treatment rate required to reduce prevalence by ¼, ½, or ¾ in Edinburgh, Melbourne, and Vancouver within 15 years (by 2027). Bars (and numbers) indicate the mean value, with whiskers representing the 95% CrI.

Minimal and achievable levels of treatment scale-up result in substantial impact in Edinburgh and Melbourne. Scaling-up treatment to 20 per 1,000 PWID annually could result in relative prevalence reductions within 15 years of 69% (95% CrI, 54%-83%) and 23% (95% CrI, 17%-32%) in Edinburgh and Melbourne, respectively, but only 9% (95% CrI, 7%-15%) in Vancouver. Higher treatment rates (>40 per 1,000 PWID annually) are required to reduce prevalence by over >20% in Vancouver within 15 years. A scale-up to treating 80 per 1,000 PWID annually could reduce HCV chronic prevalence to below 5% in Edinburgh and Melbourne, and to 30% in Vancouver, within 15 years.

Figure 4 shows the levels of treatment necessary to reduce prevalence by ¼, ½, and ¾ within 15 years (10 years after full scale-up) in all settings. Halving current prevalence could be achieved through scaled-up treatment rates of 15 (95% CrI, 12-19), 40% (95% CrI, 30-50), and 76 (95% CrI, 56-102) per 1,000 PWID annually in Edinburgh, Melbourne, and Vancouver, respectively. This would require doubling treatment rates in Edinburgh (currently 32 PWID [8 per 1,000 PWID] annually). However, in Melbourne, it would require a 13-fold scale-up (currently 75 PWID [3 per 1,000 PWID] annually), and in Vancouver would require a 15-fold scale-up (currently 68 PWID [5 per 1,000 PWID] annually). Reducing prevalence by ¾ would require a scale-up of three-fold in Edinburgh (to 22 [95% CrI, 18-27] per 1,000 PWID annually), 18-fold in Melbourne (to 54 [95% CrI, 43-67] per 1,000 PWID annually), and 20-fold in Vancouver (to 98 [95% CrI, 74-127] per 1,000 PWID annually). This would result in HCV chronic prevalences of <10% in Edinburgh, <15% in Melbourne, and <20% in Vancouver, respectively.

**Figure 5:**
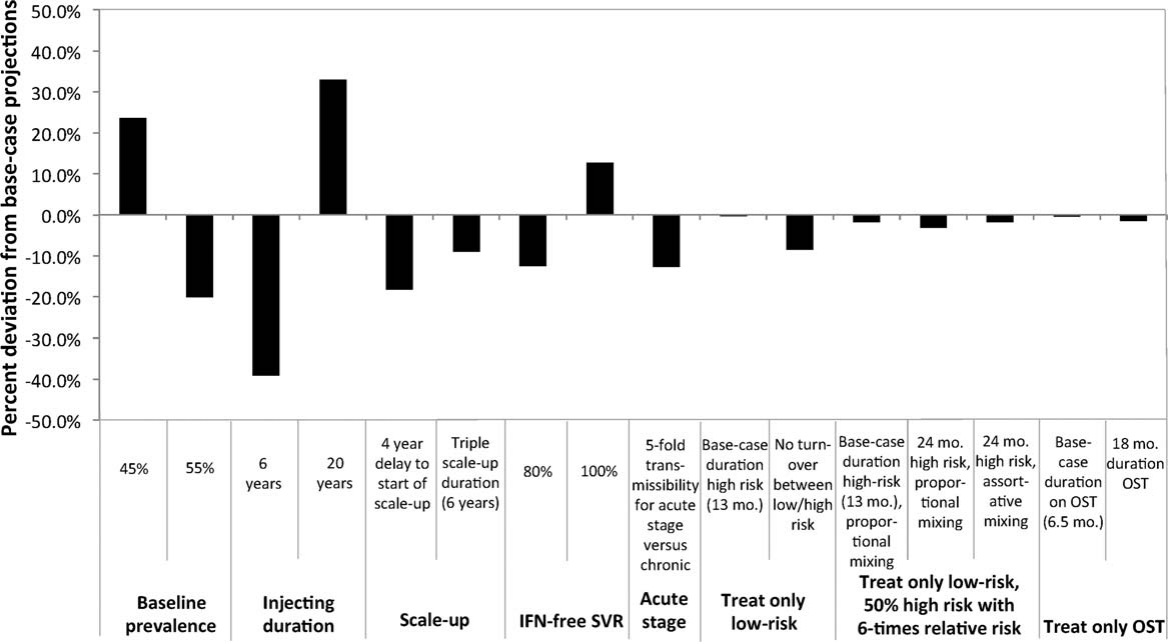
Results from the one-way sensitivity analyses; percent change from the base-case scenario of the predicted relative prevalence reduction at 15 years in Melbourne with scaled-up treatment rate of 10 per 1,000 PWID annually (from a baseline rate of 3 per 1,000 PWID annually). For the base-case, all chronically infected PWID (high/low risk or on/off OST) were eligible for treatment. Mo., months.

### Uncertainty/Sensitivity Analysis

Analysis of covariance indicated that uncertainty in average injecting duration contributed to the majority of variation (59%-78%) in impact at 15 years with a treatment scale-up to 10 per 1,000 PWID annually. The remaining variation was due to uncertainty in baseline treatment rate in Edinburgh, baseline prevalence in Melbourne, and baseline prevalence and death rate in Vancouver.

One-way sensitivity analyses showed baseline prevalence, injecting duration, and time to scale-up initiation had the most effect on model projections at 15 years with treatment scale-up to 10 per 1,000 PWID annually (Fig. 5, shown for Melbourne). Across the sites, if baseline chronic HCV prevalences were 5% lower, the impact of treatment scale-up increased by 24%-37%, whereas if baseline prevalences were 5% higher, impact decreased by 20%-27%. If the average injecting career was 20 rather than 11 years, then potential impact increased by 16%-53% (with greater impact at higher chronic prevalence), whereas if average injecting duration was shorter at 6 years, impact was reduced by 29%-43%. Delaying the initiation of scale-up by 4 years (2019 versus 2015) resulted in 7%-18% less impact. Decreasing/increasing IFN-free DAA SVR rates (to 80%/100% versus 90%) correspondingly decreased/increased impact by 12% to 15%. If acute infection was associated with a five-fold increase in transmissibility compared with chronic infection (equal for base-case), impact was reduced by 11%-16%.

Changing other assumptions regarding treatment duration or population heterogeneity (e.g., average time in OST/high risk, proportion high-risk, relative transmission risk when in OST or high-risk, mixing assumptions between low and high-risk, restricting treatment to only those on OST or low-risk) had <10% impact on projections for a scaled-up treatment rate of 10/1,000 PWID annually. However, at higher treatment rates (e.g., 80 per 1,000 PWID for Melbourne), sustaining treatment at this level would require treating the non-OST population or expanding OST coverage.

### Budgetary Impact

Previous cost-effectiveness analyses estimated the drug-only cost of triple therapy with protease inhibitors in the United States at approximately $50,000 USD per course.^40^ The cost of future IFN-free DAA regimens is unknown, but if they cost $50,000 ($25,000-$75,000), then the scaled-up treatment rates necessary to halve prevalence within 15 years (15, 40, and 76 per 1,000 PWID annually in Edinburgh, Melbourne, and Vancouver, respectively) would require an annual HCV treatment budget for PWID of $3.2 million ($1.6-$4.7 million) in Edinburgh, $50.0 million ($25-$75 million) in Melbourne, and $51.3 million ($25.7-$77.0 million) in Vancouver.

## Discussion

This modeling study explored the feasibility of HCV treatment as prevention in the era of IFN-free DAA-based HCV therapy. Current levels of HCV treatment among PWID are projected to only achieve modest or negligible reductions in HCV chronic prevalence among PWID. However, scaling up treatment could lead to substantial reductions in HCV prevalence. In Edinburgh, a doubling of treatment rates (to 15 per 1,000 PWID annually) could halve prevalence; a three-fold increase could reduce chronic HCV prevalence to <7% within 15 years. Greater scale-up will be required in Melbourne and Vancouver, where current treatment rates are lower and chronic prevalence higher, but prevalence could be halved in 15 years with treatment rates of 40 per 1,000 PWID (a 13-fold increase from 3 per 1,000 PWID annually) in Melbourne and 76 per 1,000 PWID (a 15-fold increase from 5 per 1,000 PWID annually) in Vancouver. A 20-fold increase from baseline treatment rates could reduce chronic prevalence to <15% and <20% in Melbourne and Vancouver, respectively, in 15 years.

Such scale-up, though considerable in Melbourne and Vancouver, has been achieved and exceeded for HIV treatment in both resource rich and poor settings,^41^ and even amongst PWID in some settings.^42^,^43^ In addition, programs designed to address barriers to care among PWID have achieved yearly HCV treatment rates of 40-80 per 1,000 PWID with PEG-IFN+RBV in Australia, Canada, Europe, and the United States.^44^–^47^ Moreover, scale-up of IFN-free DAA in theory will be easier to implement and have greater impact than current treatment regimes. IFN-free DAA regimens will require shorter duration and less complex monitoring^22^ which in combination with higher SVR and reduced toxicity will markedly accelerate the current expansion of HCV treatment into the community, including integration with drug treatment, such as OST.

### Limitations

These projections are based on a theoretical mathematical model, with several limitations. First, there is uncertainty in a number of parameters. These projections are predicated on assumptions of the effectiveness of IFN-free DAAs (based on phase 2 studies as evidence from large-scale evaluations are not yet available). Outcomes among PWID are unknown, but systematic reviews report similar response rates among PWID and non-PWID for IFN+RBV regimens.^48^,^49^ Additionally, active PWID are generally younger (a meta-analysis^48^ found a lower median age (38 years) for studies with HCV treatment among PWID compared with registration trials for PEG-IFN+RBV (43-45 years)) and have less advanced liver disease than the broader HCV population. We do not explicitly model HIV/HCV coinfection, as two of our settings have marginal (<1%) coinfection prevalences. However, in settings where a greater proportion of PWID are HIV/HCV-coinfected, lower SVR rates may be achieved. Sensitivity analyses revealed that a lower SVR of 80% would still achieve substantial impact, although slightly higher treatment rates would be required to achieve specific reductions in HCV prevalence.

Furthermore, better information on average injecting duration could substantially reduce uncertainty in the projections. The average age (and injecting duration) of people in drug treatment and serological surveys in the three sites suggest injecting durations between 11 and 27 years,^19^,^28^,^35^ but unbiased estimates are unavailable. An 11-year average injecting duration was assumed,^34^ but if it were longer, then greater impact would be achieved. Also, HCV risk and treatment uptake will vary between PWID subgroups, relating to injecting patterns or other factors such as homelessness. However, we considered scenarios where HCV treatment is delivered only in OST or when PWID are at low risk and show there is little impact on the outcome given movement between low-and high-risk states over an injecting career.

Second, complexities involved in treatment scale-up are not modeled. Treatment scale-up will likely be delivered in the community alongside OST, but additional interventions may be required to increase the case-finding among PWID, including health care workforce training and interventions addressing stigma surrounding testing and treatment. Importantly, in our model, a fixed number of PWID are treated annually; therefore, as prevalence falls, an increasing proportion of infected PWID are treated. This will have implications for diagnosis and treatment retention, particularly among those PWID who are more difficult to reach. However, treatment recruitment may become easier as more PWID are treated.

Third, the model assumes a stable injecting population size that, although true in the settings examined, may not be applicable to all settings. For example, data from Amsterdam^50^ suggest a decline in the number of injectors. In these settings, as PWID prevalence falls, we would expect HCV prevalence to increase as the cohort ages, and detailed models of these settings would require age-specific information on prevalence of PWID and injecting duration to determine intervention impact.

Finally, the model incorporates current levels of OST, but it did not consider the impact of scale-up or targeting of interventions such as OST and NSP, which may contribute additionally toward reducing HCV transmission.^9^ As our aim was to explore the scale-up of antiviral treatment, we did not stratify the population by drug-type or explore OST eligibility criteria. Additionally, we do not explicitly model NSP, but account for existing levels of coverage in modeling the epidemic in each setting.

### Implications and Comparison With Other Studies

This is the first analysis to explore the potential of new and future direct-acting HCV antiviral therapy as prevention in a range of global prevalence settings, and supports previous modeling studies indicating that HCV antiviral treatment could reduce transmission and HCV prevalence among PWID.^12^–^16^ In contrast, mathematical models have shown that scale-up of OST/NSP could have considerable impact in areas with historically low levels of OST/NSP; however, in many developed countries where coverage is already high (such as our sites), the scale-up required (e.g., 80% PWID on OST or high coverage NSP for 15 years) would be unachievable and unsustainable, and would achieve less impact than modest levels of HCV treatment.^9^

Overall, the projections suggest IFN-free DAA HCV treatment, as prevention is a feasible option for reducing the future burden of HCV-related disease, which is of critical public health importance given the lack of alternative effective HCV prevention strategies. HCV treatment is cost-effective, and in most settings treatment of PWID is highly cost-effective,^17^ primarily because of the potential prevention benefit and reduction in secondary transmission.

A question still remains, though, as to whether scale-up is affordable—especially if the drugs are marketed at similar cost to existing therapy. Expansion will be costly, and so any future scale-up of HCV treatment for prevention will require drug-price reform, especially for lower and middle income settings, but possibly also for developed countries that require high treatment rates.

## Abbreviations

CrI: credible interval
DAA: direct-acting antiviral
HCV: hepatitis c virus
HIV: human immunodeficiency virus
NSP: needle and syringe programs
OST: opiate substitution therapy
PEG-IFN: pegylated interferon
PWID: people who inject drugs
RBV: ribavirin
SVR: sustained viral response.
