# Supplementary material for: Hepatitis C virus treatment for prevention among people who inject drugs: Modeling treatment scale-up in the age of direct-acting antivirals
Source: Hepatology. 2013 Aug 26;58(5):1598–609. doi: 10.1002/hep.26431 (PMC3933734; doi:10.1002/hep.26431)
Supplement: Supplementary file 1 [file hep0058-1598-sd1.doc]

**SUPPLEMENTARY INFORMATION**

**Mathematical model**

We use a dynamic, deterministic, compartmental model of HCV transmission and treatment among people who inject drugs (PWID). The model included compartments for uninfecteds (*X_j,k_*), acutely infected (*A_j,k_*), chronically infected (*C_j,k_*), on antiviral treatment (*T_j,k_*), and treatment failures (F*_j,k_*). Additionally, the PWID population was stratified by risk (low/high, *j*=0 or 1, respectively), and OST (off/on, *k*=0 or 1, respectively). We track changes in the populations over time, *t*.

New injectors enter the susceptible PWID population at a rate (θ), a proportion of which (φ) enter as high risk, and the remainder (1-φ) entering as low risk. We assume all PWID are not on OST when initiating injecting, but can subsequently be recruited into OST at a rate (β). PWID remain on OST for a duration (1/γ). Low risk PWID can move to high risk at a rate (η), and remain high risk for a duration (1/κ). We fit both β and η such that the proportion on OST or high risk remains constant throughout the simulation and equal to the proportions entering each state.

Uninfected PWID can become acutely infected with HCV, where a proportion (δ, 26%[^1^](#_ENREF_1)) of individuals spontaneously clear their acute infection after a duration of time (1/ψ, approximately 0.5 years[^2^](#_ENREF_2)), and return to the uninfected compartment. Those who do not spontaneously clear the acute infection (1-δ) progress to chronically infection, where they are eligible for antiviral treatment. As PWID are unlikely to be diagnosed during acute infection, we assumed PWID are not treated during the acute stage. If treated, a proportion (α(t)) achieve SVR and return to the uninfected compartment. Those who do not attain SVR (1-α(t)) move to the treatment failure compartment. We conservatively assumed treatment failures cannot be retreated because baseline treatment rates are low, so few PWID previously treated with PEG-IFN+RBV would be eligible for retreatment with DAAs. If retreatment were allowed, the impact projections would be more than our base-case. PWID exit all compartments due to permanent cessation of drug use (μ_1_) or death due to drug or non-drug related causes (μ_2_).

We neglected immunity in the model due to the lack of strong data surrounding the presence of immunity (either following spontaneous clearance or successful treatment), and because previous analyses have shown that incorporating immunity has minimal impact on model projections.

The full model equations are as follows, for low risk PWID not on OST:


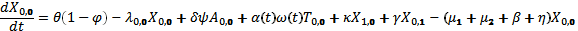


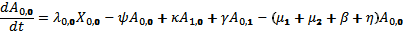


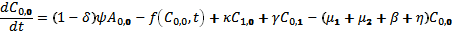


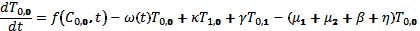


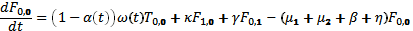


For low risk PWID on OST:


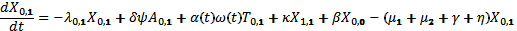


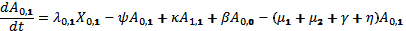


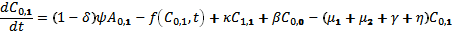


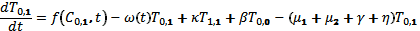


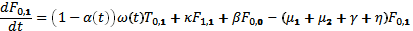


For high risk PWID not on OST:


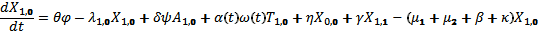


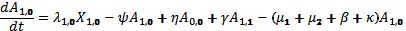


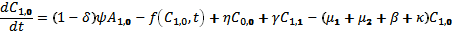


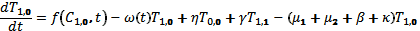


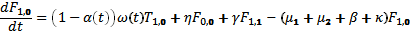


For high risk PWID on OST:


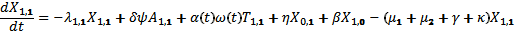


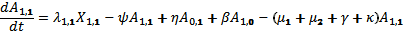


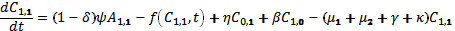


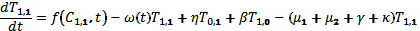


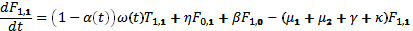


where f(*C_j,k_*, *t*) is the number of PWID initiated onto treatment per year from each chronic infection compartment, *C_j,k_*. A fixed total number (Φ(t)) of chronically infected PWID are initiated onto treatment per year in the population, with a treatment duration of 1/ω(t). Treatments are allocated proportionally to the eligible groups. If Φ(t) is greater than the number of eligible chronic infections, all eligible chronic infections are treated.

For example, if all chronically infected PWID are eligible for treatment (low/high risk and on/off OST), then


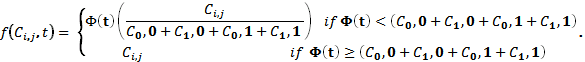


Alternatively, if only PWID on OST are eligible for treatment, then
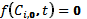
 and


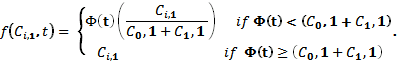


The forces of infection for each susceptible state were defined by the relative risk in that particular state, such that infectivity and susceptibility was altered by a factor Γ, Π, or Γ×Π if the PWID was on OST, high risk, or high risk and on OST, respectively. This was assumed to occur through a corresponding change in the relative frequency of transmission events with other PWID. The chance of a PWID having a transmission event with any PWID from another risk state (high/low risk or on/off OST) and infectious status was proportional to the relative frequency of transmission events for PWID in that state. Due to the rapid reduction in viral loads during treatment[^5^](#_ENREF_5), we assumed that during treatment the proportion who will eventually achieve SVR (α(t)) are not infectious, whereas the remainder (1-α(t)) remain infectious. For the base-case analysis, we assume equal transmissibility from the acute stage as compared to the chronic stage (Ξ=1), but explore increased transmissibility during the acute stage (Ξ>1) in the sensitivity analysis. The forces of infection for the base-case (assuming proportional mixing) were defined by:


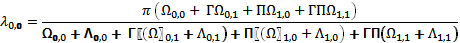


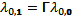


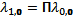


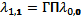


where


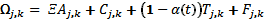


and


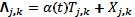
.

For the sensitivity analysis examining fully assortative mixing between low and high risk PWID, the following forces of infection were used:


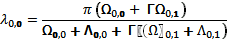


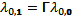


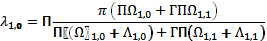


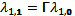


**Parameters**

Edinburgh parameters were obtained from the Needle and Exchange Surveillance Initiative (NESI) surveys 2007[^6^](#_ENREF_6), 2008/2009[^7^](#_ENREF_7), and 2010 (unpublished), and the Scottish HCV treatment database[^8^](#_ENREF_8). Melbourne estimates were taken from Melbourne injecting drug use (MIX) and Networks cohorts, and NSP survey estimates from 2002-2011. Vancouver parameters were determined from the Community Health and Safety Evaluation (CHASE) and Urban Health Research Initiative (UHRI) cohorts[^13-15^](#_ENREF_13).

*Total number of PWID*

We estimate there are 4,241 [3,276-5,681 95% CI] PWID in Edinburgh (calculated by using previous estimates [^16^](#_ENREF_16) and inflating by a factor of 1.3 based on subsequent Bayesian analysis of Scotland PWID estimates[^17^](#_ENREF_17)). Melbourne has approximately 25,000 PWID, estimated from the number of OST clients[^18^](#_ENREF_18) and self-reported OST treatment uptake. In Vancouver, the number of PWID has been estimated at 13,500 [10,000-15,000 95% CI] [^20^](#_ENREF_20).

*High risk*

We used unstable housing as a measure of ‘high-risk’, as it has been associated with HCV incidence in Vancouver (RR 1.4 [1.0-2.1, 95% confidence interval])[^15^](#_ENREF_15) and Scotland/UK (RR 3.6 [1.5-8.7, 95% confidence interval]). No population characteristics have yet been associated with HCV incidence in Melbourne. Although unstable housing has been associated with a higher risk of HCV acquisition, it was not statistically significant, possibly due to a lack of power in the study[^9^](#_ENREF_9)). We therefore assume a high risk population in Melbourne approximate in size to that of the unstable housing fraction (17% [14-20%, 95% confidence interval], unpublished data from MIX[^10^](#_ENREF_10) cohort), with the relative risk comparable to that seen in the UK (RR 3.6), as it was the highest risk ratio reported for unstable housing across the cities.

*OST*

The reduction in HCV susceptibility and infectivity on OST was determined from a recent meta-analysis of pooled UK data[^22^](#_ENREF_22), which showed a relative risk of 0.41 [0.21-0.82, 95% confidence interval] while on OST. We note that not all studies have shown this effect. For example, a meta-analysis by Hagan et al.[^23^](#_ENREF_23) found a relative risk of acquiring HCV of 0.60 (0.35, 1.03) for PWID on OST, but the effect did not reach statistical significance.

*Injecting duration*

Site-specific estimates of the average duration of injecting until long-term cessation are unavailable and difficult to obtain due to bias. Longitudinal surveys of PWID recruited from drug treatment sites tend to over-represent active injectors with a longer duration of injecting, whereas general population surveys over-represent ex-injectors with short periods of injecting. Sweeting et al. [^24^](#_ENREF_24), combined UK population surveys and other information on PWID in order to adjust for sample biases, subsequently estimating 11 years from initiation to permanent cessation. We therefore use 11 years as the average injecting duration until cessation point value in all the sites. For Edinburgh, the cross-sectional NESI survey reported a median injecting duration of 8 years (with a 32 year median age of survey participant). Other longitudinal surveys – that may be subject to recruitment bias – estimate longer average duration. For example, the Edinburgh Addiction Cohort estimated a median duration of injecting of 5 years for people unexposed to OST (with 30% ceasing within one year) and 20 years for people with longer exposure to OST[^25^](#_ENREF_25). Therefore we sample from a wide uncertainty interval for the uncertainty and sensitivity analyses.

For the UK, we sample from the range reported in Sweeting et al.[^24^](#_ENREF_24) of 6-20 years (using a triangular distribution with 11 years as the mean). For Melbourne, the median injecting duration reported in the Australian NSP survey (from which we estimated HCV prevalence) is approximately 15 years[^11^](#_ENREF_11) (with a median survey age of 35), 7 years higher than that reported in Edinburgh in the NESI survey. Hence, we increase the upper range by 7 years for Melbourne as compared to Edinburgh (sampling from 6-27 years, mode 11, triangular distribution). For Vancouver, the median injecting duration reported in the VIDUS cohort (from which we estimated HCV prevalence) was 11 years at baseline[^15^](#_ENREF_15) (with a median age of 34), hence we increase the upper sampling range by 3 years as compared to Edinburgh, sampling from 7-23 years (mode 11, triangular distribution).

*Treatment prior to 2002*

Clinical guidance in the UK, Europe, US, and Australia recommended against treatment of PWID prior to 2002[^26-29^](#_ENREF_26). Therefore, we model no treatment of PWID before 2002.

**Simulation Methods**

All equations were solved using MATLAB (version R2010a), using the inbuilt ordinary differential equation solver ODE45, a variable timestep solver based on a Runge-Kutta formula. Simulations were performed on a MacBook Pro with a 2.33 GHz Intel Core 2 Duo processor, running OS 10.7.5.

**References**
